# Cultivar-specific volatile profiles in *Passiflora edulis* determine thrips (*Frankliniella intonsa*) feeding preferences

**DOI:** 10.3389/fpls.2025.1667805

**Published:** 2025-09-11

**Authors:** Wei Li, Ziwen Ren, Tong Wang, Xiaoxia Wei, Hui Wei, Houjun Tian, Ruibin Xu, Jiyang Dong, Liangquan Wu, Biao Huang

**Affiliations:** ^1^ Research Institute of Agricultural Quality Standards and Testing Technology, Fujian Academy of Agricultural Sciences, Fujian Key Laboratory of Agro-products Quality & Safety Development & Biotechnology, Fuzhou, Fujian, China; ^2^ International Magnesium Institute, College of Resources and Environmental Sciences, Fujian Agriculture and Forestry University, Fuzhou, Fujian, China; ^3^ College of Food Science, Fujian Agriculture and Forestry University, Fuzhou, China; ^4^ Fruit Research Institute, Fujian Academy of Agricultural Sciences, Fuzhou, Fujian, China; ^5^ Fujian Key Laboratory for Monitoring and Integrated Management of Crop Pests, Fuzhou Scientific Observing and Experimental Station of Crop Pests of Ministry of Agriculture and Rural Affairs, Institute of Plant Protection, Fujian Academy of Agricultural Sciences, Fuzhou, China; ^6^ Department of Electronic Science, School of Electronic Science and Engineering, Fujian Provincial Key Laboratory of Plasma and Magnetic Resonance, State Key Laboratory of Physical Chemistry of Solid Surfaces, Xiamen University, Xiamen, China

**Keywords:** *Passiflora*, *Frankliniella intonsa*, cultivars, HS-SPME-GC-MS, volatile organic compounds

## Abstract

The issue that the attraction of leaf odors leading to thrips attacks affecting the yield and quality of Passion fruit (*Passiflora edulis*)—a high-value tropical crop in southern China has drawn widely attentions. This study aimed to comprehensively investigate the influences of special VOCs in susceptible and resistant passion fruit cultivars on feeding preferences of thrips. To explore the association between VOCs and insect behavior, the aromatic intensity of the selected cultivar leaves was determined. A total of 87 differential volatiles were identified from 423 VOCs using orthogonal partial least squares-discriminant analysis (OPLS-DA, P< 0.05, VIP > 1.0). Metabolic pathway analysis linked the differential volatiles to phenylpropanoid and α-linolenic acid metabolism. The key differential volatiles were fruity odor benzaldehyde and green odor (Z)-3-hexenol, which exhibited the highest rOAVs. Resistant cultivars accumulate high concentrations of benzaldehyde (OAV:191.49), which correlated with thrips attraction; while susceptible cultivars accumulated higher concentration of (Z)-3-Hexenol (OAV: 200.60), associated with repellency. Behavioral assays confirmed thrips preference for benzaldehyde (58% attraction) and aversion to (Z)-3-Hexenol (22% selection). These findings not only could advance our understanding of plant volatile-mediated insect behavior but also enable the development of lures for pest management, while providing a scientific basis for breeding pest-resistant cultivars.

## 1 Introduction

Passion fruit (*Passiflora edulis*) is known for its rich aroma, distinctive flavor and medicinal value. It has been commercially cultivated as a crucial economic crop in many agricultural regions (Xia et al., 2021; Li et al, 2022; Nikolova et al, 2024). Thrips (*Frankliniella intonsa*) not only negatively impact the overall health of plants and the marketability of fruit but also serve as vectors for plant viruses. Consequently, the resulting reductions in yield and fruit quality have substantial economic implications for farmers (Santos and Varon, 2012; Sultana et al, 2023; Thakur et al, 2024).

Thrips possess highly sensitive olfactory systems that allow them to detect and respond to the plant volatiles, which play an important role in foraging and locating behaviours (Zeng et al, 2024). The study revealed that these thrips were particularly attracted to benzenoids such as benzaldehyde. Changes in volatile emissions during plant growth significantly influence thrips population dynamics. Ren et al (2020) found that flowering plants attract thrips more strongly than vegetative plants due to higher concentrations of green leaf volatiles (GLVs), particularly 4-ethylbenzaldehyde, methyl cinnamate, methyl 4-ethylbenzoate, methyl benzoate and methyl salicylate, than those in the vegetative state. Moreover, plant volatile substances can also repel thrips. A study investigated the behavioral responses of four thrips species to volatiles emitted by rosemary (*Rosmarinus officinialis* L.) and found that its repellent effect may be attributed to two major compounds, α-pinene and eucalyptol, as demonstrated in both olfactometer bioassays and cage experiments (Li et al, 2021b). These studies emphasize the critical role of specific volatiles in influencing insect behavior. However, despite these findings, our understanding of the compounds responsible for attracting or repelling thrips feeding on passion fruit remains limited.

Plant-derived volatile organic compounds (VOCs) function as essential signaling mediators that orchestrate defense activation through three interconnected metabolic pathways (Loreto et al, 2014). The phenylpropanoid pathway biosynthesizes methyl salicylate (MeSA), a phytohormonal derivative that primes systemic acquired resistance (SAR) through epigenetic reprogramming of defense genes and coordinated accumulation of pathogenesis-related proteins, thereby conferring durable broad-spectrum immunity against microbial pathogens (Gong et al, 2023). Complementing these mechanisms, the lipoxygenase (LOX) pathway rapidly converts linolenic acid into green leaf volatiles (GLVs) through hydroperoxide lyase (HPL)-mediated catalysis, generating characteristic C6 aldehydes, alcohols, and esters. Despite comprehensive characterization of VOC-mediated defenses in model angiosperms, the ecological stoichiometry and molecular regulation of these pathways in passion fruit remain enigmatic, particularly their co-evolutionary dynamics with tropical herbivores and microbial communities under field conditions.

In the present study, we analyzed leaf volatile organic compounds (VOCs) from thrips-resistant (MY) and susceptible (QM) passion fruit cultivars. By comparing their VOC profiles, we identified key compounds that differentially regulated thrips attraction, validated through selective olfactory responses. Further metabolic mapping linked these compounds to phenylalanine biosynthesis and fatty acid metabolism pathways, revealing that enhanced (Z)-3-Hexenol production in QM serves as a critical resistance trait, whereas MY’s heightened thrips susceptibility was associated with elevated benzaldehyde accumulation. This integrated approach clarifies how metabolic divergence shapes cultivar-specific thrips resistance.

## 2 Materials and methods

### 2.1 Plant materials and growth conditions

Two passion fruit cultivars, MY (purple passion fruit, susceptible to thrips) and QM (yellow passion fruit, high thrips-resistant), were provided by the Fruit Research Institute of Fujian Academy of Agricultural Sciences in Fuzhou, China. They were cultivated to maturity in a field located in Fujian, China (25°89’N, 119°58’E), from March to November 2024. The experimental design incorporated a flat shed plus curtain planting method, featuring a row spacing of 1.0 m by 2.0 m, a height of 1.8 m, a ridge height of 40 cm, and a ridge width of 1 m, with a planting density of 450 plants per ha. Nitrogen, phosphorus, and potassium balanced fertilizer (N:P_2_O_5_:K_2_O = 15:15:15) was applied prior to transplanting. Root water was administered immediately after transplanting. Liquid fertilizer was applied every 15 days. A 20% tefuran suspension and a 5% imidacloprid aqueous dispersion were sprayed every 7 days before shelf placement. Pesticides were sprayed every 15 days post-shelf placement.

### 2.2 Sample collection

Passion fruit leaves were collected for the experiment in the climbing stage of passion fruit growth (April 6, 2024). The selected plants were all transplanted at the same time, exhibited uniform growth, reached a height of approximately 1.5 m, and were free from pests and diseases. From each plant, three to five of the topmost intact and healthy leaves were collected around 10 a.m. in the morning. Leaves from every three plants form one replicate sample, with six replicates established for each cultivar. Leaves from each of the three replicates were gently wiped with alcohol to remove surface impurities, then immediately frozen in liquid nitrogen and stored at -80°C for volatile organic compounds analysis.

Field collection was conducted by gently tapping passion fruit leaves over a white porcelain tray to dislodge thrips specimens, which were then carefully aspirated into 1.5 mL microcentrifuge tubes using a handheld aspirator. The collected thrips were immediately preserved in 70% ethanol for morphological examination. Under a stereomicroscope, the thrips exhibited typical Thysanoptera characteristics (Leica K8, Germany).

### 2.3 Metabolome analysis

Volatiles were analyzed with a GC-MS system (QP2020 NX, Shimadzu, Japan) equipped with a DB-5MS column (30 m × 0.25 mm × 0.25 μm, Agilent Technologies, USA). Firstly, the leaves were quickly frozen using liquid nitrogen and subsequently ground into a fine powder. 0.5 g of a powder of leave was transferred into a 20 mL headspace vial containing 2 mL NaCl, then 20 μL of 3-Hexanone (10 μg mL-1, CAS: 589-38-8) was added into vial as internal standard (Xia et al, 2021). The vial was sealed by PTFE−silicone septum (VFAP-606050B-18M-100, Anpel) immediately, and incubated at 60°C for 5 min. The extraction head was conditioned at 250°C for 15 min in the Fiber Conditioning Station before absorbing the volatiles. A SPME probe coated with 50/30 μm DVB/CAR/PDMS (Agilent Technologies, USA) was exposed to the head-space for 15 min at 60°C to extract the volatiles.

Then the SPME probe was introduced into the GC injection port and desorbed at 250°C for 5 min. The carrier gas was helium (purity ≥ 99.999%) delivered at a constant flow rate of 1.2 mL min-1. The front inlet mode was splitless, and the solvent delay was 3 min. The column temperature ramp was as follows: 1) the initial oven temperature was 40°C, held for 3.5 min; 2) raised to 100°C at a rate of 10°C min-1; 3) raised to 180°C at a rate of 7°C min-1; 4) raised to 280°C at a rate of 25°C min-1, held for 5 min. The mass conditions were set as follows: the ion source temperature was 230°C, and the interface temperature was 280°C. Electron ionization (EI) mode was 70 eV with a scanning range of 35-550 m z-1. SIM mode was scan.

GC-MS raw data were processed using MassHunter Quanlitative Analysis Software (version B.10.00, Agilent Technologies, USA) to perform peak picking, deconvolution, and compound annotation. Tentative identification of volatiles was performed by comparison of the database from the NIST 2014 library, the retention index (RI, determined by n-alkanes C7-C40) and the experimentally determined. The chemical composition of the volatile substances was determined, and the compound content was calculated by a semi-quantitative method (normalized peak area). The volatiles were quantitated in terms of the internal standards (3-hexanone) by a semiquantitative method. The mean and standard deviation of compound concentrations are calculated and expressed as mean ± SD. These data were used to reveal the relationship among samples by principal component analysis (PCA). Cluster heat map analysis was performed on all samples using R package of Complex Heatmap. Differential metabolites were screened based on variable importance in projection (VIP) ≥ 1 using partial least square-discrimination analysis (PLS-DA), fold Change (|log_2_FC|) >1, and p< 0.05 (BH-adjusted t-test). Differential metabolites were mapped to the Kyoto Encyclopedia of Genes and Genomes (KEGG) databases (http://www.kegg.jp) to identify enriched metabolic pathway.

Absolute quantification was employed to determine the concentrations of benzyl alcohol, benzaldehyde, (Z)-3-hexenol, (E)-2-nonenal, and (E)-2-hexenal in the leaves of both varieties. These reference material (purity ≥ 99%, GC grade) were obtained from Yuanye Bio-Technology Co., Ltd. (Shanghai, China). The relative/absolute content of volatiles was calculated based on Liang et al. (2024).

### 2.4 Odor activity value calculation

Compounds with an OAV > 1 are generally considered to contribute to the aroma of passion fruit, while those with an OAV > 10 are key aroma components. Calculate the OAV of each component of the two cultivars, the OAV was calculated as follows:


$$\text{OAV}=\frac{C_{i}}{OT_{i}}$$

Where $C_{i}$ is the compound concentration ( ${\text{mg kg}}^{-1})$ , and $OT_{i}$ is the olfactory detection threshold in water ( ${\text{mg kg}}^{-1})$ .

### 2.5 Selective olfactory responses of *Frankliniella intonsa* to differential volatiles

The selection behavior of *Frankliniella intonsa* adults in response to plant volatiles was evaluated using a glass Y-tube olfactometer (arm length: 8 cm; internal diameter: 1 cm; angle between arms: 75°). Each arm was connected in series to a sample chamber, a flow meter, a humidified air purification system (containing activated charcoal and distilled water scrubbers), and an air pump. The activated charcoal filter removed airborne contaminants to prevent interference with volatile profiles, while the water humidifier maintained stable humidity and further purified the air.

Prior to testing, target volatiles were dissolved in n-hexane (1 µg mL^-1^), and 20 µL of each solution was applied to filter paper and placed in one arm of the olfactometer. The control arm contained a cotton leaf disc treated with 20 µL of n-hexane alone. Adult thrips were starved for 2-3 h before being released individually at the entrance of the Y-tube.

Behavioral observations were performed for 5 min per insect. A positive response was recorded when a thrips crossed the midline of either olfactometer arm and maintained its position for ≥15 s; trials where no such movement occurred were categorized as ‘no response’. Between experimental batches (10 replicates each), the olfactometer was thoroughly rinsed with 75% ethanol, air-dried, and rotated 180° to control for directional bias.

Behavioral responses of Frankliniella intons to different volatile treatments were evaluated using attraction rate and repellency rate as assessment indices. The formulas were calculated as follows:


$$\text{Attraction rate}\;\left(\%\right)=\frac{t}{t+c}\times \;100\;$$

$$\text{Repellency rate}\;\left(\%\right)=\frac{c}{t+c}\times \;100\;$$

where t represents the number of individuals selecting the treatment arm, and c denotes those choosing the control arm.

### 2.6 Statistical analysis

All experimental data were expressed as the mean ± SD of triplicate analyses. Analysis of variance (ANOVA) was used to test the significance, followed by comparisons of two cultivars using Tukey’s test (*p*< 0.05). Statistical analyses were performed using SPSS Statistics ver.24 (IBM, 2016). The HCA (hierarchical cluster analysis) results of samples and metabolites were presented as heatmaps with dendrograms, while Pearson correlation coefficients (PCC) between samples were calculated and presented in *R*. Both HCA and PCC were carried out by *R* package of Complex-Heatmap. For HCA, normalized signal intensities of metabolites (unit variance scaling) are visualized as a color spectrum.

## 3 Results

### 3.1 Field phenotypes of thrips resistance in two passion fruit cultivars

**Figure 1:**
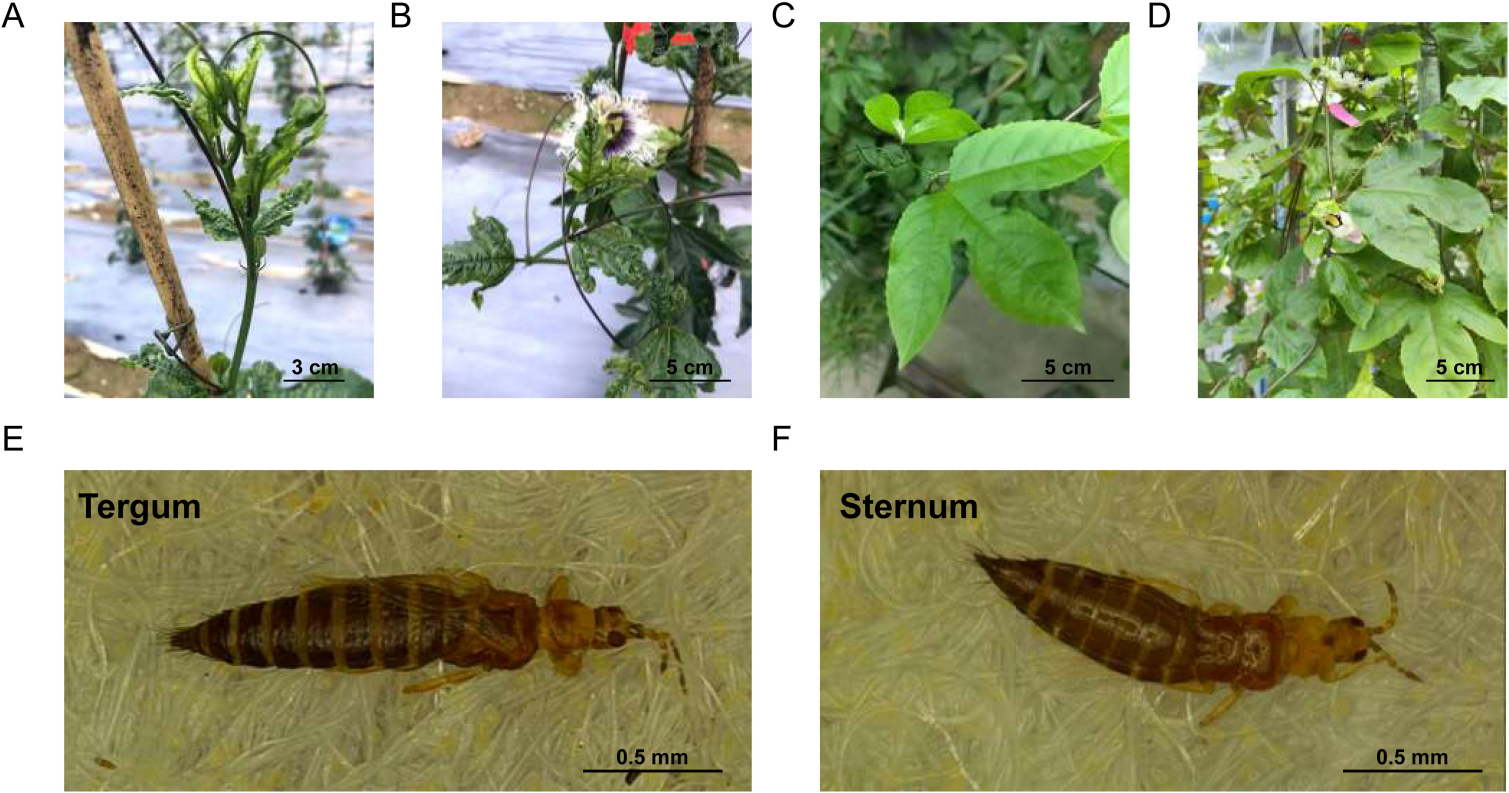
Field phenotypes of thrips resistance in two passion fruit cultivars during vine development stage. The damage symptoms on MY (susceptible) tender branches **(A)** and leaves **(B)** that were fed on by thrips. QM (resistant) was almost untouched by thrips, with its tender branches **(C)** and leaves **(D)** showing no damage. The tergum **(E)** and sternum **(F)** of *Frankliniella intonsa* are shown in the respective panels.

We observed the feeding behavior of thrips on two passion fruit plants in the field during vine development stage (April of spring), when the thrips population reached its peak. The tender shoot and leaves of MY (susceptible) exhibit characteristic damage symptoms, including stunted growth, distortion, and curling. In severe cases, there can be necrotic spots or areas where the tissue has died, leading to a general decline in leaf health and appearance (**Figures 1A, B**). Conversely, QM (resistant) does not seem to be favored by thrips, as its leaves exhibit a healthy, intact appearance with no visible signs of damage or discoloration and retain their natural green color and smooth surface (**Figures 1C, D**).

We isolated *Frankliniella intonsa* adults from infested passion fruit leaves collected in the field. The thrips exhibited typical morphological characteristics of this pest species, with slender yellowish-brown bodies measuring approximately 1.2-1.5 mm in length. The adults displayed narrow fringed wings that appeared grayish under stereomicroscopy, with distinct dark setae along the wing margins. Microscopic examination revealed the characteristic eight-segmented antennae, with segments III-IV bearing forked sense cones—a key diagnostic feature for this species. The body surface showed fine sculpture and microtrichia, while the abdominal tergites exhibited paired campaniform sensilla and posterior margins with craspedum (toothed projections) (**Figures 1E, F**).

### 3.2 Identification of VOCs in leaves of two passion fruit cultivars

**Figure 2:**
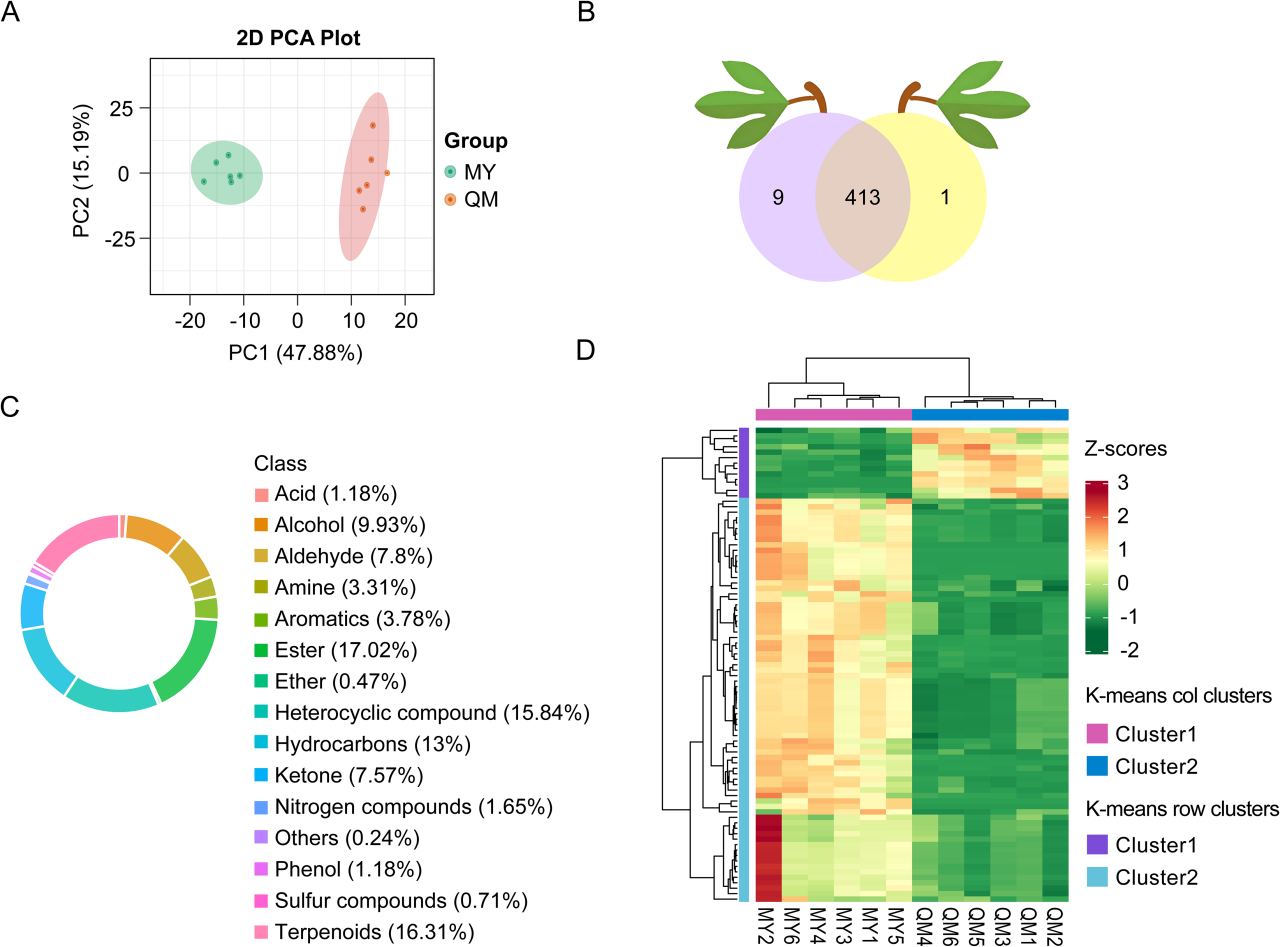
Composition and principal components of VOCs in the leaves of two passion fruit cultivars. **(A)** PCA score plot with each point representing an independent biological replicate. **(B)** The number of volatiles detected in the leaves of two passion fruit cultivars. **(C)** Circular chart of the composition proportions of volatile compound categories. **(D)** Cluster heat maps of Z-score standardized VOCs in the leaves of two passion fruit cultivars. Clustering is calculated using the Euclidean correlation and average linkage.

To elucidate thrips’ leaf feeding preferences between two passion fruit cultivars, we profiled VOCs emitted by intact, healthy leaves using HP-SPME-GC-MS. PCA of the relative peak area (**Figure 2A**) revealed two well-separated clusters corresponding to QM and MY, with no sample overlap—underscoring the reproducibility of our HS-SPME-GC-MS workflow. The two cultivars showed a primary separation along PC1, which accounted for 47.88% of the total variation, indicating there were significant differences in the VOCs of the two cultivars of leaves. After deconvolution and library matching against the NIST database (matching score > 85%), we annotated 423 distinct VOCs: 414 in QM and 423 in MY (**Figure 2B**). These volatiles spanned 15 chemical classes, dominated by esters (17% of total abundance) that confer sweet-fruity notes, and terpenoids (16.3%) responsible for the characteristic floral-citrus aroma of passion fruit (**Figure 2C**). Hierarchical clustering of normalized VOC abundances partitioned 413 common metabolites into two distinct clusters (**Figure 2D**). Cluster 1, enriched in QM, and Cluster 2, enriched in MY. Together, these results indicated clear cultivar-specific volatile signatures, both in composition and relative abundance, that likely contribute to the observed differences in thrips feeding behavior.

### 3.3 Differential VOCs screen between two cultivars

**Figure 3:**
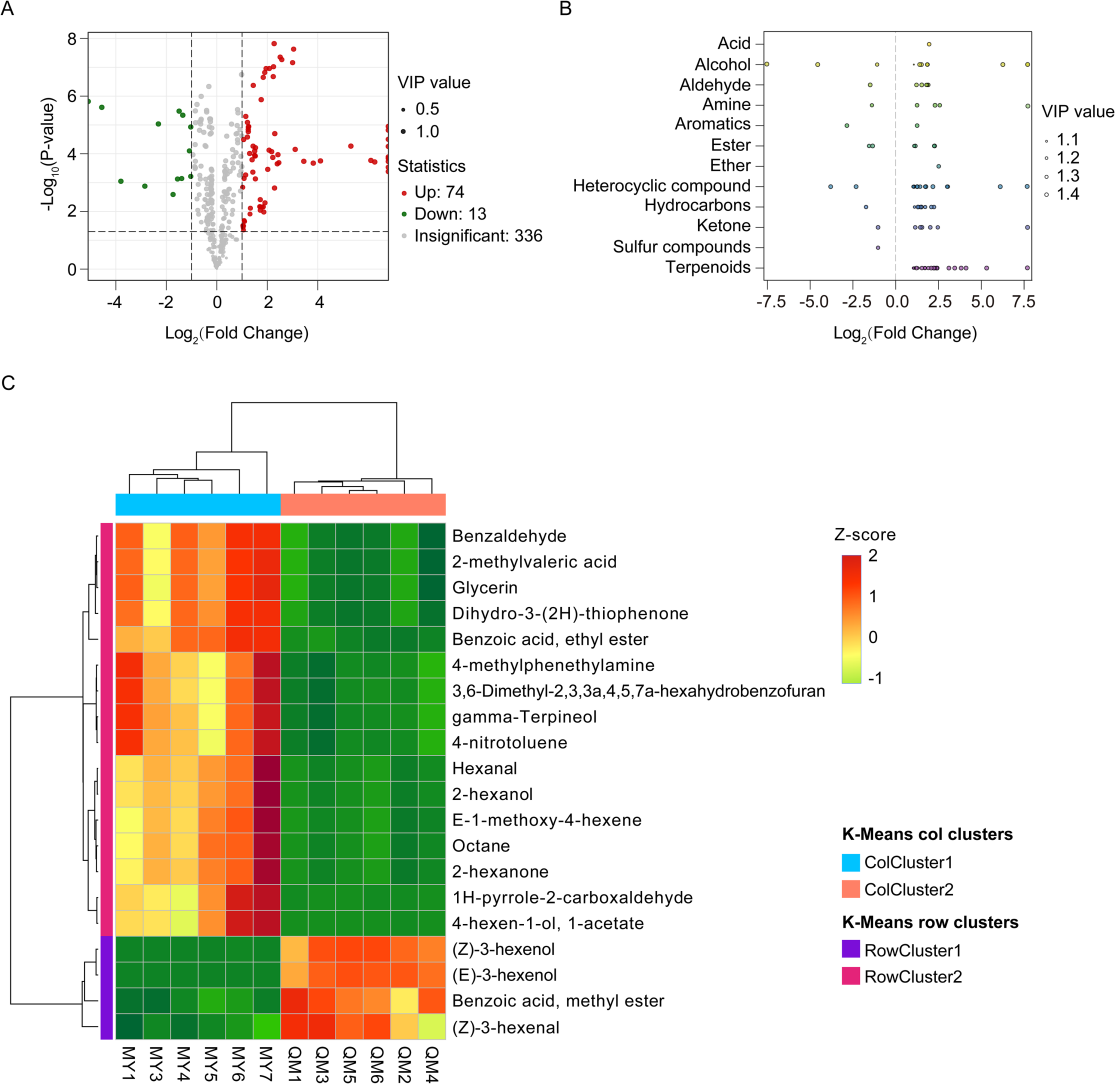
Differences in volatile accumulation among the two cultivars. **(A)** Volcano plots of differential VOCs. **(B)** Scatter plot of differential VOCs categories. **(C)** Hierarchical cluster analysis of top 20 differential VOCs. Green represents downregulated metabolites, red represents upregulated metabolites, and gray represents metabolites having no significant differences. The Z-score method based on the relative abundance mean and SD of the original data was used to standardize the differential VOCs content.

Differential VOCs were screened based on variable importance in projection (VIP>1) of PLS-DA model, fold changes (|log_2_FC|>1), and statistical significance (*p*< 0.05) of t-test with BH adjusted. We identified a total of 87 differential VOCs, with 74 being upregulated and 13 downregulated in MY compared to QM (**Figure 3A**). Among the differential VOCs identified, terpenoids were the most abundant, comprising 25% (**Figure 3B**). Within the terpenoid class, lavandulol and β-ocimene stood out as exhibiting the greatest differences (**Supplementary Table 1**). These compounds are known to significantly contribute to the vanilla flavor in passion fruit leaves. Among the aldehyde compounds, benzaldehyde showed the most significant variations. Benzaldehyde often associated with sweetness, which contributes to the intense floral and fruity aromas, are key contributors to the characteristic intense aroma and sweetness of passion fruit leaves (**Supplementary Table 1**). The significant changes in these compounds suggest their importance in shaping the sensory profile of the leaves and potentially influencing insect behavior.

**Figure 3C** presents the top 20 differential VOCs ranked by their abundance, all of which exhibit significantly different levels between the two cultivars. Aldehydes, alcohols, esters, and ketones exhibit higher relative abundances in MY. Notably, benzaldehyde stands out as the most abundant differential VOCs, with their levels in MY significantly higher than in QM. Characterized by its strong aromatic odor, benzaldehyde imparts a distinctive almond and cherry flavor to the leaves of MY. Additionally, hexanal and 1H-pyrrole-2-carboxaldehyde are also significant contributors, imparting notes of fresh grass, fruit, and nuts. Alcohols, including glycerol, gamma-terpineol and 2-hexanol, are present in higher abundance in the leaves of purple passion fruit, enhancing their fruity and floral aromas. Furthermore, two ester compounds—benzoic acid, ethyl ester and 4-hexenol acetate—as well as two ketone compounds—2-hexanone and Dihydro-3-(2H)-thiophenone—also contribute to the enhanced fruity aroma profile of MY. Thereby enhancing the complexity and appeal of the fruit’s aroma. These compounds all enhance the complexity and appeal of the aroma in MY leaves. Among the top 20 volatiles ranked by relative abundance, only four compounds were more abundant in QM than in MY. Of these, (Z)-3-Hexenol, (E)-3-Hexenol, and methyl benzoate—key contributors to green and fruity-floral aromas—exhibited significantly higher concentrations in QM leaves compared to MY (**Figure 3C**). This compound has a fresh, grassy aroma with herbal and vegetal notes that enhance the unique grassy flavor of QM leaves. While it enriches the aroma, higher concentrations of these volatiles might also increase perceived bitterness, which could potentially deter thrips.

Generally, the overall source of aroma in the leaves of MY exhibited a higher abundance of volatiles. Compared to QM, these compounds were present in greater quantities in MY, playing a crucial role in defining its unique and complex aromatic characteristics. The high abundance of these aromatic substances not only enhances MY’s distinctive appeal to thrips but also highlights the uniqueness of this variety in terms of its olfactory attributes.

### 3.4 Functional assessment of aroma components from differential VOCs in the leaves of two cultivars

**Figure 4:**
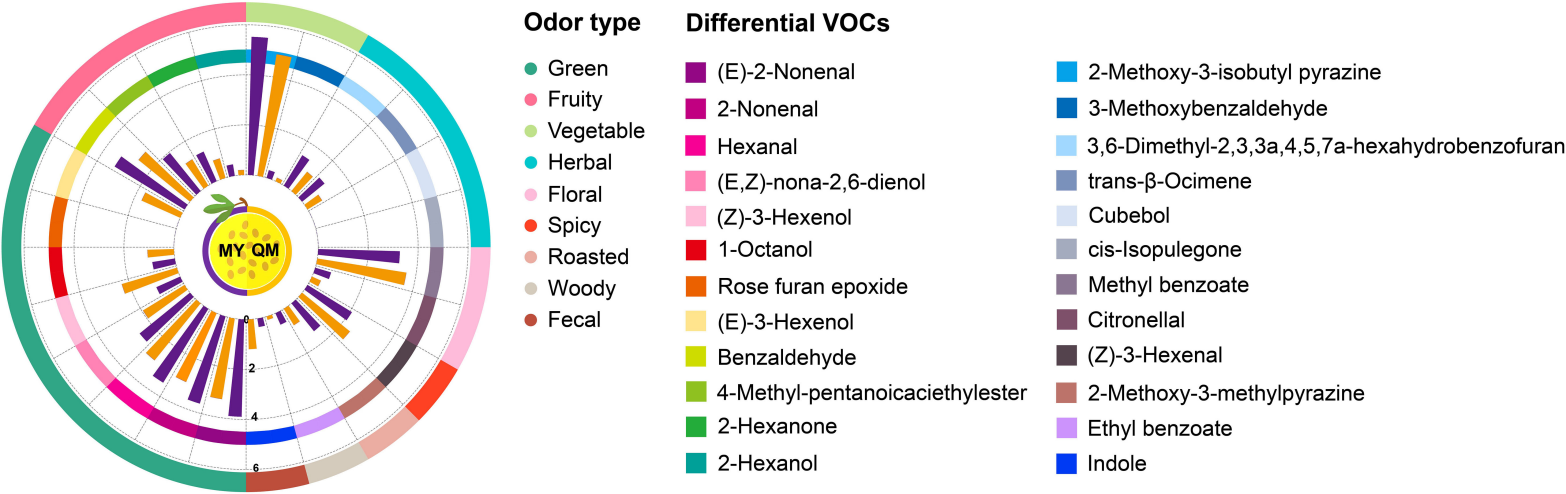
Differences in aroma component intensity of leaves of two cultivars. Different colored trapezoids represent the various VOCs. Purple and orange bars represent the odor intensity of various VOCs in MY and QM, respectively. The trapezoid of the outer ring denote distinct aroma characteristics. The trapezoid of the inner ring denotes the odor intensity of differential VOCs. The Log_10_ (mean + 1) method based on the mean of the original data was used to standardize the odor intensity of differential VOCs. Odor descriptions were mainly gathered from an open source website: www.flavornet.org/flavornet.html.

Despite the infrequent direct application of the term odor activity value (OAV) in entomological studies, the fundamental concept of threshold-dependent activity assessment is extensively utilized. This approach involves evaluating the significance of volatile organic compounds based on their concentrations relative to species-specific olfactory thresholds, thereby providing insights into their potential ecological roles and impacts on insect behavior (Dweck et al, 2018). The impact of differential VOCs on the feeding behavior of thrips among two cultivars of leaves were assessed by incorporating the OAV of these compounds (Li et al, 2023). **Table 1** lists the content and OAVs of 24 differential VOCs with aromatic characteristics, which were identified from among a total of 87 differential VOCs. Of these, 18 substances have higher concentrations in MY compared to QM, whereas 6 substances are more abundant in QM than in MY. The MY leaves did not contain detectable levels of (E)-3-hexenol but did contain cubebol, rose furan epoxide, and cis-isopulegone, compounds that were absent in QM. These distinct chemical profiles serve as important markers for differentiating between the MY and QM (**Figure 4** and **Table 1**).

**Table 1:** Differences in aroma component intensity of leaves of two cultivars.

No.	Compounds	Content (mg kg ^-1^)		Odor threshold* (mg kg ^-1^)	OAV	
		MY	QM		MY	QM
1	2-Hexanone	1.83	0.54	0.098	18.67	5.51
2	(Z)-3-Hexenal	0.48	1.15	0.004	120.00	287.50
3	Hexanal	14.50	4.29	0.005	2900.00	858.00
4	Citronellal	0.21	0.09	0.06	3.50	1.50
5	Ethyl benzoate	3.05	0.54	1.43	2.13	0.38
6	2-Hexanol	2.86	0.86	1.5082	1.90	0.57
7	trans-β-Ocimene	0.44	0.13	0.034	12.94	3.82
8	(E)-2-Nonenal	0.62	0.15	0.00008	7750.00	1875.00
9	3,6-Dimethyl-2,3,3a,4,5,7a-hexahydrobenzofuran	0.75	0.27	0.03	25.00	9.00
10	(E,Z)-nona-2,6-dienol	0.39	0.09	0.001	390.00	90.00
11	Methyl benzoate	0.97	2.10	0.00052	1865.38	4038.46
12	(Z)-3-Hexenol	0.50	10.03	0.05	10.00	200.60
13	Benzaldehyde	671.47	154.35	0.35	1918.49	441.00
14	4-Methyl-pentanoicaciethylester	0.53	0.10	0.006	88.33	16.67
15	2-Methoxy-3-methylpyrazine	0.08	0.01	0.003	26.67	3.33
16	(E)-3-Hexenol	0.00	5.30	0.11	0.00	48.18
17	1-Octanol	0.15	0.23	0.022	6.82	10.45
18	2-Nonenal	0.43	0.10	0.0001	4300.00	1000.00
19	2-Methoxy-3-isobutyl pyrazine	0.66	0.17	0.000002	330000.00	85000.00
20	3-Methoxybenzaldehyde	0.20	0.07	0.181	1.10	0.39
21	Indole	0.05	0.58	0.04	1.25	14.50
22	Cubebol	0.03	0.00	–	–	–
23	Rose furan epoxide	0.04	0.00	–	–	–
24	cis-Isopulegone	0.03	0.00	–	–	–

MY and QM represent two cultivars, respectively.

The leaves of MY and QM passion fruit primarily exhibit green pepper and fresh grass aromas. Among these, the green compounds contributing most significantly to the green aroma intensity are (E)-2-Nonenal, followed by 2-Nonenal, Hexanal, (E,Z)-nona-2,6-dienol, (Z)-3-Hexenol and 1-Octanol. Only (Z)-3-Hexenol and 1-Octanol exhibit higher contents and aroma intensities in QM compared to MY. Conversely, the other five VOCs are present at 3 to 4-fold higher levels in MY, contributing to its more pronounced green flavor. Benzaldehyde, 4-Methyl-pentanoicaciethylester, 2-Hexanone, and 2-Hexanol are present at higher concentrations in MY, with their contents and OAVs being 3 to 5-fold higher than in QM. These compounds significantly contribute to the distinctive fruity flavor profile of MY (**Figure 4** and **Table 1**).

Among these compounds, benzaldehyde is the most abundant, accounting for the highest concentration among the 24 detected VOCs. Its elevated levels are hypothesized to impart a more intense fruity aroma to MY, potentially making it more attractive to thrips for feeding. The MY contains elevated levels of 2-Methoxy-3-isobutyl pyrazine and 3-Methoxybenzaldehyde, which collectively impart distinct herbal notes evocative of fresh vegetables and thyme. The MY contains 5 to 6-folds higher levels of 2-Methoxy-3-methylpyrazine and ethyl benzoate compared to the QM, which these compounds associated with caramelized sugar and maple syrup impart a sweeter aroma. Noteworthily, (Z)-3-Hexenal and indole are found in higher contents and exhibit greater OAVs in QM. These compounds, which impart spicy and fecal odors respectively, may contribute to the repellent effect against thrips (**Figure 4** and **Table 1**).

### 3.5 Biosynthetic pathways for differential VOCs in the leaves of two cultivars

**Figure 5:**
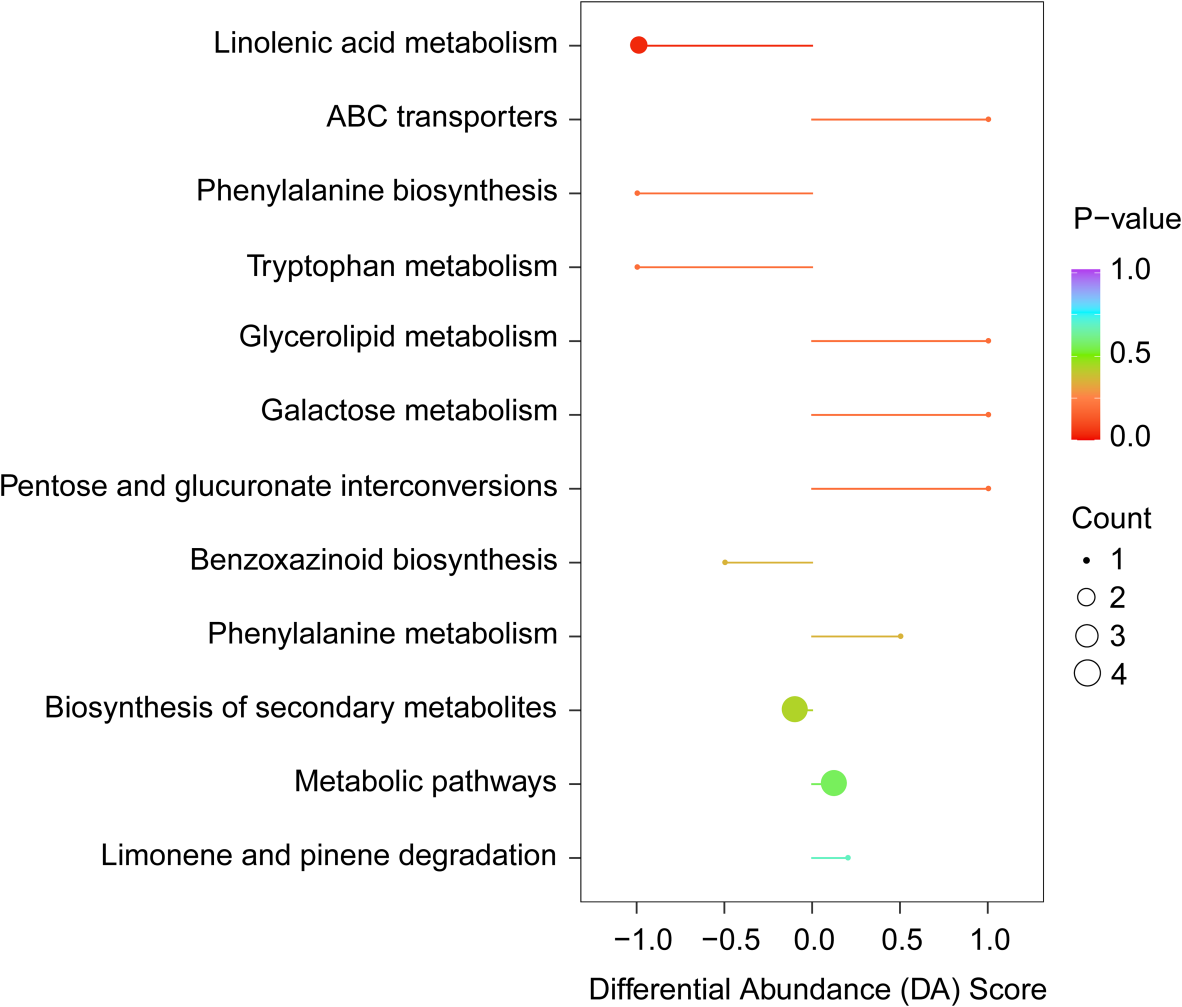
Pathway-based differential abundance (DA) analysis reveals differences in metabolic traits between two cultivars. A DA score > 0 indicates that the detected metabolites involved in pathway increase in MY relative to QM, whereas a DA score< 0 indicates that the detected metabolites involved in the pathway decrease in MY compared to QM.

Metabolic enrichment analysis was conducted on KEGG pathway database using 87 differential VOCs. The results revealed that these metabolites are primarily associated with nine metabolic pathways, including linolenic acid metabolism, ABC transporters, phenylalanine biosynthesis, tryptophan metabolism, glycolipid metabolism, galactose metabolism, pentose and glucuronate interconversions, benzoxazinoid biosynthesis and phenylalanine metabolism (**Figure 5**). Pathway-based differential abundance (DA) analysis reveals differences in metabolic traits between MY and QM. Compared to QM, MY leaves display significant downregulation in several key metabolic pathways, such as fatty acid metabolism and phenylalanine/tyrosine/tryptophan biosynthesis. This dysregulation suggests that these pathways may significantly influence thrips feeding behavior. The absolute value of the DA-score, indicated by the length of the line segments, shows that pathways farther from the central axis exhibit more significant differences. As a result, we found that changes in these pathways, particularly those involving phenylalanine degradation and α-linolenic acid metabolism, could impact thrips feeding patterns (**Figure 5**).

**Figure 6:**
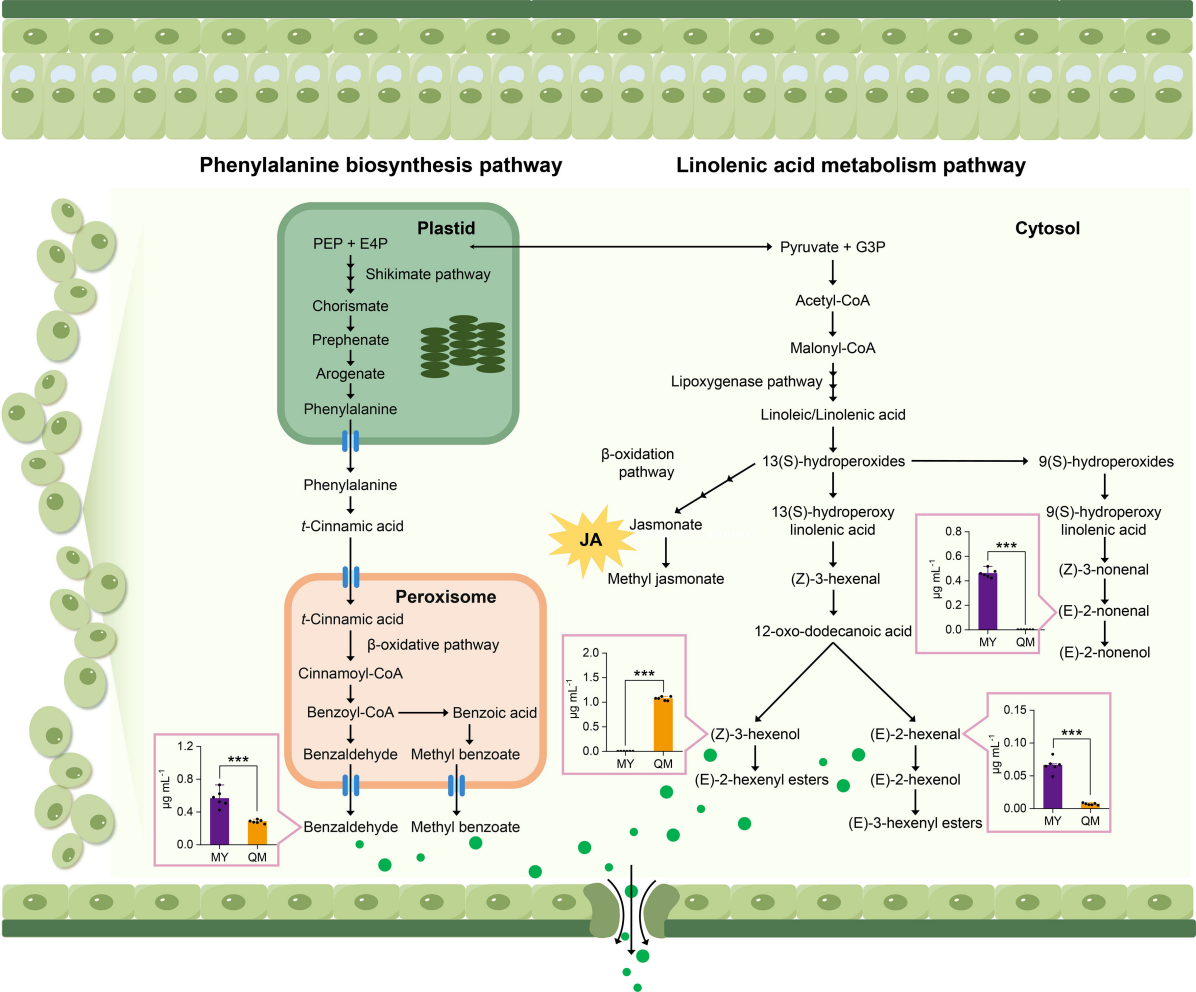
Biosynthetic pathways for key flavor volatiles. Green circles represent GLVs. Multiple arrows represent multiple enzymatic steps. PEP, phosphoenolpyruvic acid; E4P, erythrose 4-phosphate; G3P, glyceraldehyde 3-phosphate. The histograms were drawn based on absolute contents, with the concentration of each compound expressed.

To explore the key pathways that contribute to the difference in aroma between the leaves of two cultivars, we performed absolute quantification analysis on several differential VOCs. The results showed that the benzaldehyde content in the leaves of MY was 2-fold higher than in QM, with an average concentration of 0.56 μg mL^-1^. This suggests that MY leaves have a more pronounced sweet aroma, potentially making them more attractive to thrips. In contrast, there was no significant difference in benzyl alcohol content between the two varieties, despite its relatively high concentration. This suggests that benzyl alcohol may have a limited influence on thrips feeding behavior (**Figure 6A**). Linolenic acid, serving as a precursor to n-3 fatty acids, undergoes enzymatic reactions to generate green leaf volatiles (GLVs). These compounds not only impart a fresh, grassy fragrance to plants but also trigger defense mechanisms in response to insect feeding. We quantified three characteristic VOCs in the α-linolenic acid metabolic pathway and found that QM leaves contain high levels of (Z)-3-Hexenol. As a second messenger in plant defense, the high content of (Z)-3-Hexenol may explain why QM leaves are less attractive to thrips (**Figure 6B**). Conversely, relative to QM, MY leaves contain (E)-2-Nonenal at content 10-fold higher, and (Z)-2-Hexenol was detected exclusively in MY leaves. The elevated levels of these two compounds may potentially induce thrips feeding behavior.

### 3.6 Olfactory behavioral selection of *Frankliniella intonsa* in response to two volatiles

To investigate the effects of (Z)-3-Hexenol and benzaldehyde concentrations in passion fruit leaves on the olfactory-mediated behavioral responses of *Frankliniella intonsa*. Statistical analysis revealed highly significant differential responses, *Frankliniella intonsa* adults displayed pronounced repellency to (Z)-3-Hexenol (selection rate 22%, χ²=16.67, *p<* 0.01), consistent with known avoidance behaviors to green leaf volatiles at ecologically relevant concentrations. Conversely, a marked attractant response was observed toward benzaldehyde (58% selection, χ²=20.17, *p<* 0.01), suggesting this aromatic aldehyde may function as a kairomone in thrips-host interactions. The 2.6-fold preference ratio (benzaldehyde vs. (Z)-3-Hexenol) provides quantitative evidence for volatile-specific orientation behavior in this agronomically significant pest species.

## 4 Discussion

### 4.1 Metabolomics reveals interspecific differences in volatiles of passion fruit leaves

There are considerable differences in the distribution of aroma components in the tissues of different varieties of passion fruit (Pontes and Marques, 2009; Zheng et al, 2024). This study found that the main volatile compounds in passion fruit leaves were esters (17%) and terpenoids (16.3%), supporting previous findings regarding their contribution to aromatic profile of the leaves (Li et al, 2021a). The accumulation of volatiles was quite different in the leaves of MY and QM. We identified a total of 87 differential VOCs, of which 74 were more abundant and 13 were less abundant in MY compared to QM (**Figure 3A**). Among these, benzaldehyde was the most abundant aldehyde, with its absolute content being 2-fold that of QM. This compound formed the basis of the sweet almond-like aroma characteristic of leaves of purple passion fruit. Glycerol is the most abundant alcohols in MY, with twice the relative abundance found in QM, and it influences volatility and sensory experience by interacting with other flavor compounds, thereby changing the olfactory threshold. Glycerol’s impact on the volatility of these compounds increases with its concentration (**Figure 3C**) (Wang et al, 2024). (Z)-3-Hexenol were present in high relative abundance in QM, imparting a fresh green aroma to the QM leaves (**Table 1**). High levels of (Z)-3-Hexenol contribute a spicy scent to the QM leaves. The aroma component intensity for leaves showed that the intensity of (Z)-3-Hexenol and indole are 10 to 20 folds higher in QM compared to MY. At high intensities, indole can have a fecal odor (Kim et al, 2021). Each passion fruit cultivar exhibits a unique volatile profile, purple passion fruit predominantly contains hexyl butyrate, while yellow passion fruit is characterized by methyl hexanoate, (Z)-β-ocimene identified as the signature aromatic compound in banana passion fruit (Pontes and Marques, 2009). Comparative metabolomics reveals cultivar-specific terpenoid allocation in passion fruit—yellow passion fruit (*P. edulis f. flavicarpa*) accumulates taste-modulating terpenoids, while purple passion fruit (*P. edulis Sims*) enriches aroma-related compounds (Zheng et al, 2024). Among other berry plants, green leaf volatiles (GLVs) such as (E)-2-hexen-1-ol, 1-hexanol, and hexanal serve as significant markers for distinguishing grape cultivars (Yue and Ju, 2023). Furthermore, specific volatile compounds can serve as markers for assessing plant stress capacity (Holopainen, 2004; Fernandes and Correia, 2015).Generally, the VOC analysis provides a scientific basis for understanding the biochemical mechanisms underlying the distinct aroma characteristics of MY and QM cultivar. The identified potential VOCs indicators can facilitate cultivar identification, supporting breeding programs aimed at improving aromatic qualities.

### 4.2 Plant volatiles can influence thrips feeding behavior in plant-thrips interactions

The interspecific variation in leaf volatiles between MY and QM is a key determinant of thrips feeding preference. Green leaf volatiles act as chemical signals for herbivorous insects, enabling them to identify and locate suitable host plants from afar (Turlings and Erb, 2018). Previous research showed that Adult female western flower thrips (*Frankliniella occidentalis*) were attracted by the benzenoids benzaldehyde in a Y-shaped glass tube olfactometer. Simpson et al (2011) reports on four different field experiments that assess the potential of six plant volatiles, including benzaldehyde and (E)-3-Hexenol, to attract insects. The results indicated that thrips display a positive response to concentrations of 0.5% and 1.0% (v/v, mixed with a vegetable oil adjuvant) for both compounds in four plants. Thrips are one of the most common and particularly difficult pests to control in passion fruit cultivation (Salamanca et al, 2015). Nonetheless, there has been a lack of research specifically investigating the metabolic differences in insect resistance among various passion fruit cultivars so far.

**Figure 7:**
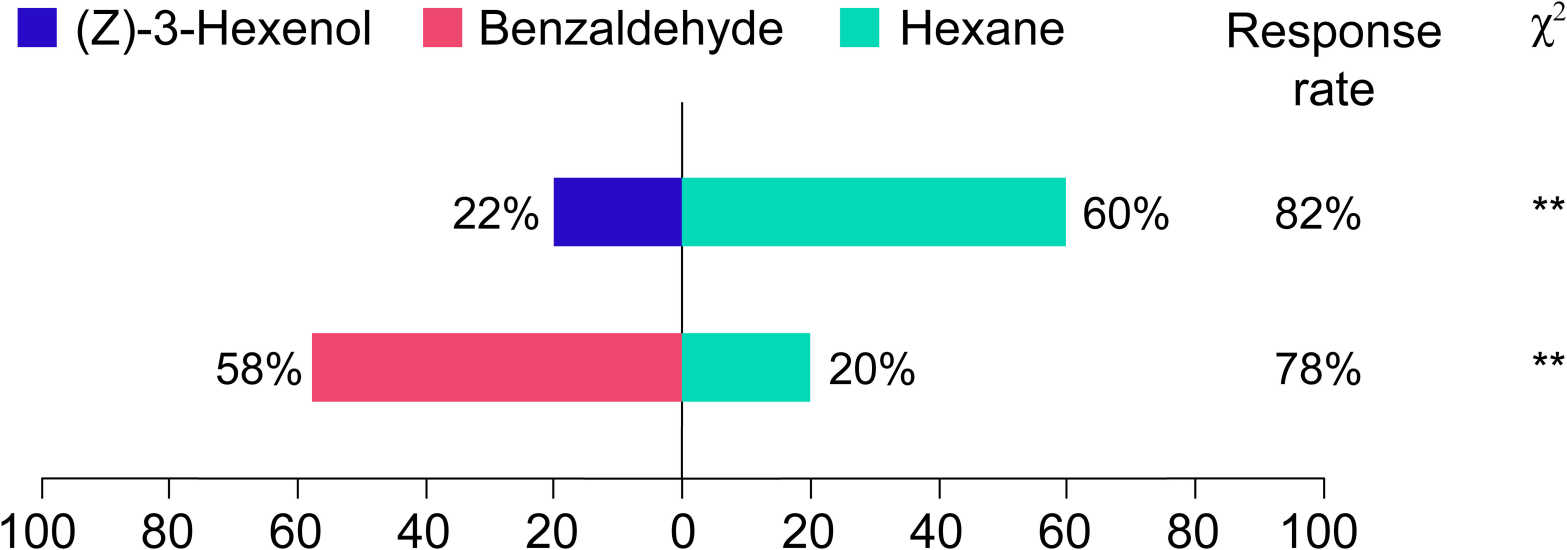
Olfactory responses of *Frankliniella intonsa* to volatiles. All test volatiles (Hexane, (Z)-3-Hexenol, and Benzaldehyde) were prepared at identical concentrations of 1 μg mL^-1^.

In this study, a total of 423 volatile compounds were identified in the leaves of Miyu, significantly more than in QM (**Figures 2B, D**). Studies have shown a significant positive correlation between the levels and number of volatiles and soluble sugar content. Since plant phloem sap and cells are rich in carbohydrates and other compounds, they are particularly favored by thrips. So, it could be inferred that the higher quantity and content of volatiles in MY were likely due to the elevated levels of carbohydrates in its leaves. These components not only facilitate the production of a larger number of volatiles but also serve as attractants for thrips, promoting their feeding behavior. Low contents of sugar and other compounds in QM leaves could reduce their attraction to thrips and indirectly improve cultivar resistance (Li et al, 2021b). We also compared the volatiles of leaves of the QM (high insect-resistant) and MY (susceptible) cultivars and quantified their differential volatiles using targeted metabolomics. Insect-resistant QM leaves were found to be highly enriched in volatiles related to the enhancement of insect resistance, with metabolic pathways mainly focused on phenylpropane biosynthesis pathway and fatty acid metabolic pathway (**Figure 5**). Benzenoids/phenylpropanoids, the second most diverse group of plant volatiles, play crucial roles in attracting or repellent insects (Lv et al, 2024). Based on both identification and semi-quantitation, MY leaves were found to contain higher levels of benzaldehyde with strong insect-repelling activity, while (Z)-3-Hexenol, a repellent compound, is nearly absent (**Figures 3C**, **6**, **7**; **Table 1**). This suggested that in the leaves of the purple passion fruit cultivars exemplified by MY, benzaldehyde was mainly synthesized through peroxisome-localized β-oxidation pathways, while the high insect-resistant of QM may be attributed to the significant enrichment of regulate defense gene expression and defense metabolite accumulation in flavonoid metabolism, then enhance QM resistance (Chen et al, 2024).

High content of (E)-2-Hexenal and (E)-2-Nonenal were identified in the leaves of MY, which were absent in QM (**Figure 6A**). According to Yuan et al (2024), behavioral assays demonstrated that female insects showed a significant attraction to (E)-2-Nonenal, which led them to lay eggs on new leaves or wounds near the apical growing part of plant. This behavior enables the larvae to access and consume the internal tissue fibers of the plant after hatching, which accounts for the severe infestation observed in MY thrips populations and can’t be treated with pesticides (**Figure 1**). Simultaneously, this could activate jasmonic acid (JA) and ethylene (ET) signals through synthesis of (E)-2-Hexenol in MY leaves to enhanced direct and indirect MY resistance reducing the performance of thrips (**Figure 3C**,**Figure 6** and **Table 1**) (Zhang et al, 2024). Pathway-based DA-score analysis showed QM leaves display significant up-regulation in ABC transporters (**Figure 5**). Synthesized in nutritional organs such as mesophyll and epidermal cells, VOCs traverse the cytoplasm with the aid of ABC transporters, eventually passing through the cuticle before exiting the cell into the intercellular air spaces connected to stomata, from where they are released into the environment (Penuelas, 2004). Adebesin et al (2017) discovered the first ABC transporter, *PhABCG1*, that can transport volatiles, including methyl benzoate. Methyl benzoate, in addition to exerting direct toxicity on pests, demonstrates considerable potential as a larval repellent and an oviposition deterrent for adult insects (Zhao et al, 2022). Therefore, we speculate that a homologous gene of *PhABCG1* may also exist in passion fruit, and QM may have higher gene expression. This could lead to increased thrips resistance by synthesizing methyl benzoate to activate the JA signaling.

### 4.3 Proposed model of insect resistance mechanisms mediated in passion fruit

**Figure 8:**
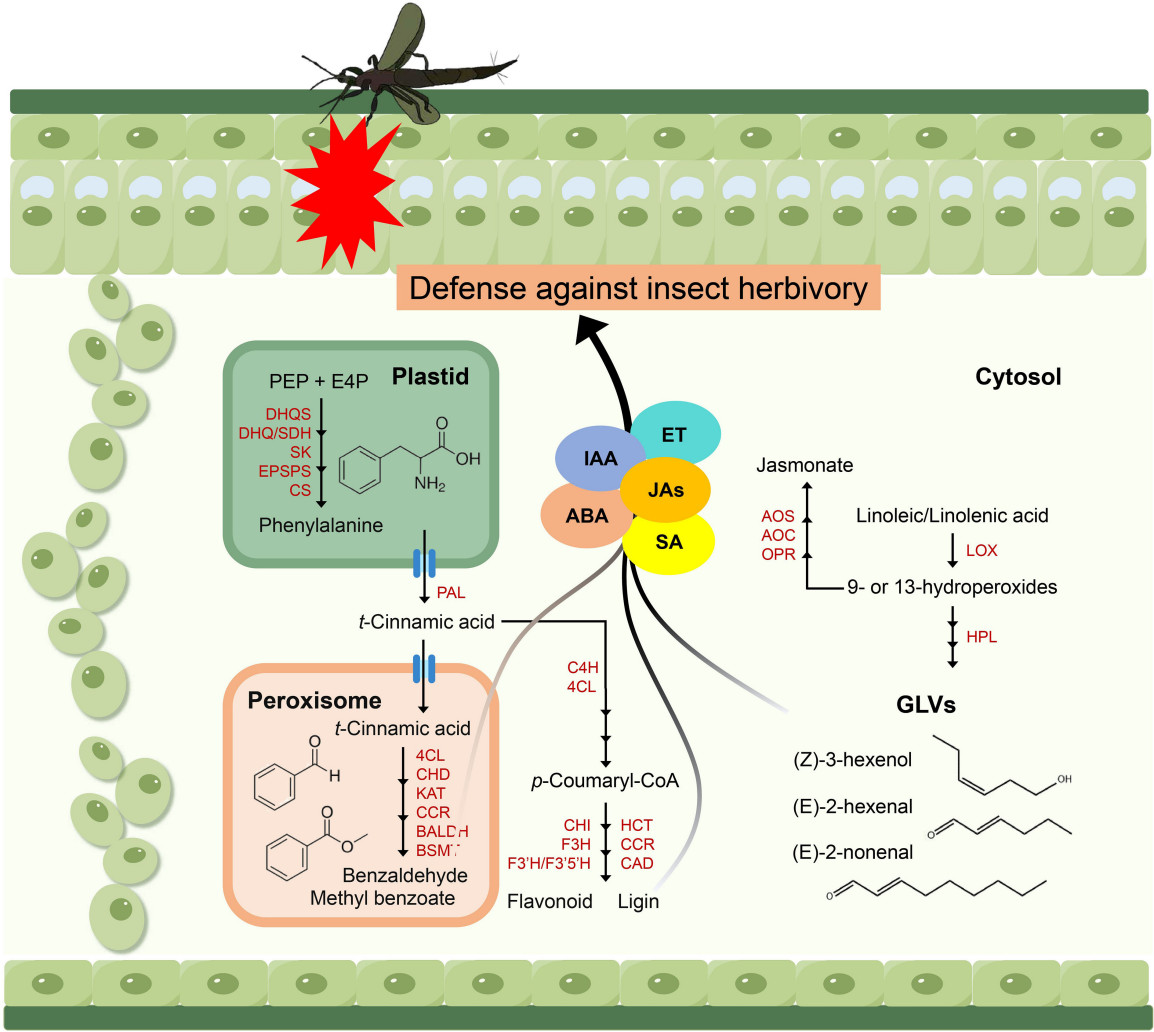
Proposed model of passion fruit insect resistance mediated by specific metabolites. DHQS, 3-dehydroquinate synthase; DHQ/SDH, 3-dehydroquinate dehydratase/shikimate 5-dehydrogenase; SK, shikimate kinase; EPSPS, 5-enolpyruvylshikimate 3-phosphate synthase; CS, chorismate synthase; PAL, phenylalanine ammonia lyase; 4CL, 4-coumarate-CoA ligase; CHD, cinnamoyl-CoA hydratasedehydrogenase; KAT, 3-ketoacyl-CoA thiolase; CCR, cinnamoyl-CoA reductase; BALDH, benzaldehyde dehydrogenase; BSMT, benzoic acid/salicylic acid carboxyl methyltransferase; C4H, cinnamate-4-hydroxylase; CHI, chalcone isomerase; F3H, flavanone 3-hydroxylase; F3’H/F3’5’H, flavonoid 3’- hydroxylase/flavonoid 3’ 5’-hydroxylase; HCT, hydroxycinnamoyl-CoA: shikimate hydroxycinnamoyl transferase; CAD, cinnamyl alcohol dehydrogenase; AOS, allene oxide synthase; AOC, allene oxide cyclase; OPR, 12-oxophytodienoate reductase; LOX, lipoxygenase; HPL, hydroperoxide lyase. Multiple arrows represent multiple enzymatic steps.

Elucidating the biosynthetic pathways of defensive aromatic compounds not only provides a theoretical foundation for understanding plant anti-insect mechanism and plant-insect interactions, but also facilitates molecular breeding of insect-resistant crops by identifying key metabolites (Gatehouse, 2002; Pichersky and Gershenzon, 2002; Lv et al, 2024). In this study, we employed metabolomics approaches to identify several key metabolic pathways and specific metabolites that are intricately linked with the defensive responses of passion fruit against insect herbivory (refer to preceding sections). By synthesizing our experimental findings with insights from the existing literature, we propose a theoretical framework that elucidates the potential process by which these specific metabolites mediate insect resistance in passion fruit (**Figures 7**, **8**).

Firstly, resistance generated in passion fruit is dependent on enhanced production of phenylalanine-derived benzenoid phenylpropanoid volatiles and fatty acid-derived volatiles. Thrips repeatedly preferred MY leaves over QM, mainly reflected in the physiological and biochemical changes of benzaldehyde and (Z)-3-Hexenal content (**Figure 6**), which involves multiple key enzymes. Studies have shown that when plants are attacked by insects, genes associated with plant defense, such as those involved in the biosynthesis of phenylpropanoids and fatty acid derivatives, such as *CCR* and *LOX*, are upregulated to resist pest damage and to trigger defense responses in neighboring plants (Guo et al, 2018). Secondly, volatiles can also induce changes in early signaling, defense genes, and defense metabolite levels. For example, GLVs can activate JA, SA, IAA, ET and ABA signaling and the expression of related genes, thereby enhancing defensive signaling pathways because they share a common substrate, there exists potential for substrate competition or feedback effects (Xin et al, 2019). In addition, GLVs can trigger synergistic or antagonistic interactions between different plant signaling cascades and metabolites, thereby enhancing plant defense through this mechanism (Erb, 2018). It is important to note that in our study, assessments were conducted on healthy leaves. Therefore, we speculate that the high resistance of QM to thrips may be due to the high expression and enhanced transcriptional activity of genes related to the composition volatiles synthesis pathway, and the synergistic interaction with the plant defense signaling pathway.

## 5 Conclusion

In conclusion, our findings indicated that (Z)-3-hexenal and benzaldehyde in passion fruit leaves can significantly regulate thrips feeding behavior. (Z)-3-hexenal exhibited repellent effect to thrip, while benzaldehyde showed significant attraction. Although metabolomics has generated pivotal insights into how green leaf volatiles mediate pest behaviors, subsequent investigations should prioritize the systematic identification and functional validation of master regulatory genes governing both fatty-acid metabolism and the phenylpropanoid pathway, thereby providing the genetic framework required for precision breeding programs.

## Data Availability

The original contributions presented in the study are included in the article/**Supplementary Material**. Further inquiries can be directed to the corresponding author.

## Supplementary material

Supplementary Figure 1 Total ion chromatograms.

Supplementary Data Sheet 1 Relative abundance of differential volatile metabolites.
