# Supplementary material for: Cultivar-specific volatile profiles in Passiflora edulis determine thrips (Frankliniella intonsa) feeding preferences
Source: Front Plant Sci. 2025 Sep 11;16:1667805. doi: 10.3389/fpls.2025.1667805 (PMC12460472; doi:10.3389/fpls.2025.1667805)
Supplement: Supplementary Figure 1 — Total ion chromatograms. [file Table1.docx]

Supplementary Material

**Supplementary Figure 1.** Total Ion Chromatograms.
